# Neuraminidase-1 (NEU1): Biological Roles and Therapeutic Relevance in Human Disease

**DOI:** 10.3390/cimb46080475

**Published:** 2024-07-26

**Authors:** Jingxia Du, Hanqi Shui, Rongjun Chen, Yibo Dong, Chengyao Xiao, Yue Hu, Nai-Kei Wong

**Affiliations:** 1 College of Basic Medicine and Forensic Medicine, Henan University of Science and Technology, Luoyang 471023, China; shq080700@163.com (H.S.); 17716392921@163.com (Y.D.); xcy0822@126.com (C.X.); 15603861842@163.com (Y.H.); 2 Clinical Pharmacology Section, Department of Pharmacology, Shantou University Medical College, Shantou 515041, China; 21rjchen@stu.edu.cn

**Keywords:** neuraminidase-1, drug development, sialic acids (SAs), disease mechanisms, lysosomal sialidase

## Abstract

Neuraminidases catalyze the desialylation of cell-surface glycoconjugates and play crucial roles in the development and function of tissues and organs. In both physiological and pathophysiological contexts, neuraminidases mediate diverse biological activities via the catalytic hydrolysis of terminal neuraminic, or sialic acid residues in glycolipid and glycoprotein substrates. The selective modulation of neuraminidase activity constitutes a promising strategy for treating a broad spectrum of human pathologies, including sialidosis and galactosialidosis, neurodegenerative disorders, cancer, cardiovascular diseases, diabetes, and pulmonary disorders. Structurally distinct as a large family of mammalian proteins, neuraminidases (NEU1 through NEU4) possess dissimilar yet overlapping profiles of tissue expression, cellular/subcellular localization, and substrate specificity. NEU1 is well characterized for its lysosomal catabolic functions, with ubiquitous and abundant expression across such tissues as the kidney, pancreas, skeletal muscle, liver, lungs, placenta, and brain. NEU1 also exhibits a broad substrate range on the cell surface, where it plays hitherto underappreciated roles in modulating the structure and function of cellular receptors, providing a basis for it to be a potential drug target in various human diseases. This review seeks to summarize the recent progress in the research on NEU1-associated diseases and highlight the mechanistic implications of NEU1 in disease pathogenesis. An improved understanding of NEU1-associated diseases should help accelerate translational initiatives to develop novel or better therapeutics.

## 1. Introduction

**Figure 1:**
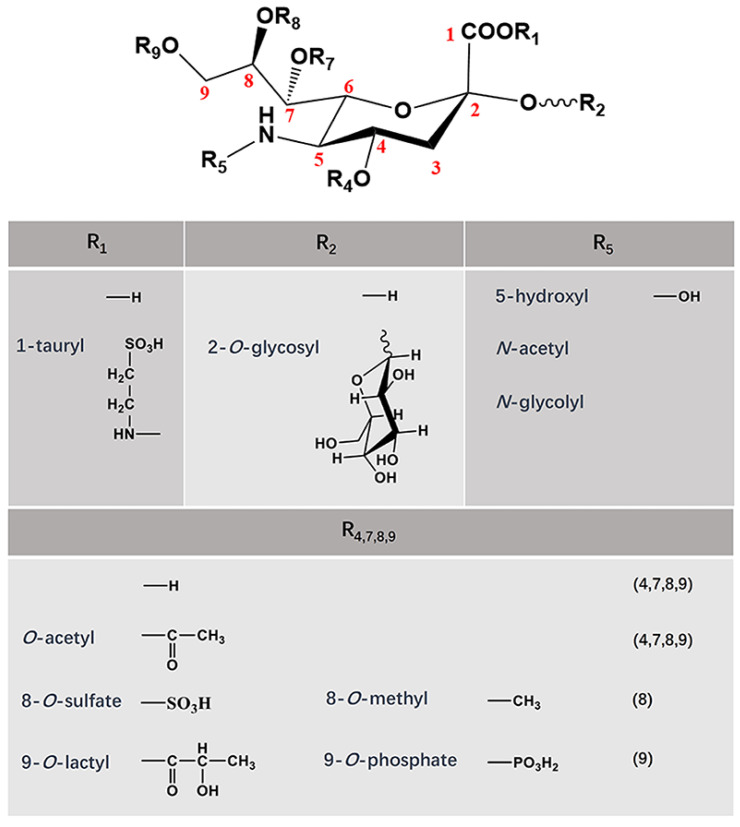
Structures and possible modifications of sialic acids [14,15,16,17].

Sialic acids (SAs) occur as a class of sugar compounds with a characteristic negatively charged nine-carbon scaffold and are widely distributed among living organisms [1]. Historically, SAs were named after their original site of discovery, the salivary gland mucin, by the Swedish biochemist Gunnar Blix [2]. Structurally, the analogs of SA are classified according to different linkage groups on the fifth carbon, with more than 50 natural derivatives being confirmed to this date, including the most abundant *N*-acetylneuraminic acid (Neu5Ac), the non-human *N*-glycolylneuraminic acid (Neu5Gc), and deaminoneuraminic acid and their single or multiple *O*-acetyl derivatives (Figure 1) [3,4]. In terms of modifications, SAs are mainly linked to galactose (Gal) or *N*-acetylgalactosamine (GalNAc) on a glycoconjugate via *α*-2,3 or *α*-2,6 glycosidic bonds (See detailed reviews in [5,6,7]). They can also be assembled into polysialic acid chains via *α*-2,8 glycosidic bonds. By virtue of their tissue and cell specificities, mammalian SAs are physiologically responsible for various biological functions, some of which are related to apoptosis and the differentiation of myeloid cells [8] as well as cellular infiltration and metastasis. In addition to participating in the regulation of developmental processes, SAs also serve as the targets of enzymatic actions against certain viral and pathogenic microbial receptors, thus affecting the dynamics and outcomes of immunological interactions [9,10,11,12]. For example, during Leishmania protozoa infection, NEU1 interacts with siglec-E to dually regulate the activation of TLR4 signaling via SA [13].

Broadly speaking, the enzymes governing SA metabolism are functionally categorized into two main groups: sialyltransferases (ST), which catalyze the covalent linkage of sialic acid to a glycoconjugate; and neuraminidases (Neus), also known as sialidases, which cleave terminal neuraminic, or sialic, acid residues from glycoconjugates. Neus encompass a broad range of exoglycosidases (EC 3.2.1.18) found in diverse species, including bacteria, viruses, protozoa, birds, and mammals (see detailed reviews in [11,18]). They are responsible for hydrolyzing terminal SA residues in glycolipids, oligosaccharides, and glycoproteins, which is a first step toward their catabolism. It has been suggested that all the Neus descend from a common ancestor, which accounts for the observed similarities of active sites in human Neus and viral Neus, for example. The best-studied Neus include viral Neu, which can serve as a drug target for limiting the spread of influenza infections. There are four principal types of Neus occurring in mammals, namely: Neuraminidase-1 (NEU1), neuraminidase-2 (NEU2), neuraminidase-3 (NEU3), and neuraminidase-4 (NEU4), which display differential yet overlapping patterns of tissue expression, cellular/subcellular localization, substrate specificity, and biological function (see a detailed review in [19]) (Table 1). The roles of Neus in human health and disease have been well recognized for their etiological implications in various conditions encompassing sialidosis [20], galactosidosis [21], neurodegenerative disorders [22], cancer [23], cardiovascular diseases [24,25,26,27], diabetes [28,29], and lung diseases [30]. Neus have thus emerged as potential therapeutic targets in various disease contexts.

Among Neus, NEU1 has been a subject of intensive study. Widely expressed in multiple systems, NEU1 plays important roles in various biological processes including lysosomal catabolism, and the selective modulation of cellular receptors. Despite sharing some general mechanisms of action, it is observed that the other three Neus cannot compensate for the lack of NEU1 in patients. Therefore, a better understanding of the biological roles and cellular dynamics of NEU1 in both physiological and pathological processes is crucial to rational conceptualization for disease treatment. This review aims to summarize recent advances in NEU1 research by highlighting its regulatory roles in related signaling pathways. In so doing, we hope to galvanize interest in the discovery and development of innovative treatment strategies for diseases linked to NEU1 dysregulation.

## 2. Principles of NEU1 Action

### 2.1. NEU1 Biological Function

NEU1 differs from other mammalian salivary enzymes in that in the lysosome, NEU1 activity requires the formation of a lysosomal multienzyme complex (LMC) comprising cathepsin A (CTSA; PPCA), *N*-acetyl-galactosamine-6-sulfate sulfatase (GALNS), and *β*-galactosidase (*β*-Gal) [39]. PPCA operates as a prerequisite transporter protein in the complex, especially for NEU1. PPCA acts additionally as a molecular chaperone, facilitating the folding, stabilization, oligomerization, and activation of NEU1. The resultant complex fully constitutes the catalytic activity of NEU1. When PPCA is uncoupled from NEU1, this results in the dissociation of the complex and NEU1 inactivation [40].

**Figure 2:**
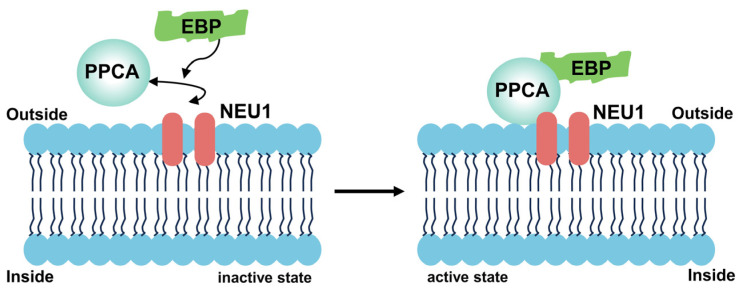
Scheme showing interactions between the components of the NEU1-EBP-PPCA complex.

In addition to the PPCA/NEU1/*β*-GAL triple-enzyme complex, another related enzyme complex, termed the elastin receptor complex (ERC) or CSER, has been identified on the plasma membrane (PM) of diverse human cell types. Functionally essential to elastogenesis, ERC comprises three major components: PPCA, NEU1, and elastin-binding protein (EBP) (Figure 2). EBP is a splicing variant of *β*-Gal that shares substantial amino acid sequence homology with the *β*-Gal enzyme but is catalytically inactive and does not localize to lysosomes. Pharmacologically, Neus inhibitors can block the assembly of elastin fibers in human skin fibroblasts, aortic smooth muscle cells, and osteocytes. In human skin fibroblasts, NEU1 deficiency in aortic smooth muscle cells causes impaired elastic fiber synthesis, which can be experimentally reversed by the overexpression of exogenous NEU1 [41,42].

### 2.2. NEU1 Substrates

NEU1 also acts as a lysosomal sialidase catalyzing the removal of terminal SAs from SA conjugates. The target of NEU1 is a glycoprotein with *α*-(2,3) and *α*-(2,6) SA linkages [43,44], whereas the activity of this enzyme is diminished against ganglioside substrates [45].

Several glycoproteins are shear-cutting substrates for NEU1, which include the cell-surface glycoprotein epidermal growth factor receptor (EGFR) [46], Toll-like receptor 4 (TLR4) [43], insulin receptor (IR) [47], integrin *β*4 [48] and CD31 [49], CD36 [50], intracellular glycoprotein lysosomal-associated membrane protein 1 (LAMP1) [51], amyloid precursor protein (APP) [22], TLR7 and TLR9 [52], etc. NEU1 orchestrates cell behavior by altering the SA modifications of these glycoproteins, thus partaking in the regulation of the relevant signaling pathways. Another NEU1 substrate present in human cells is the protein differentiation group 31 (CD31), which mediates the process of angiogenesis by undergoing desialylation to regulate endothelial cell responses [49].

**Figure 3:**
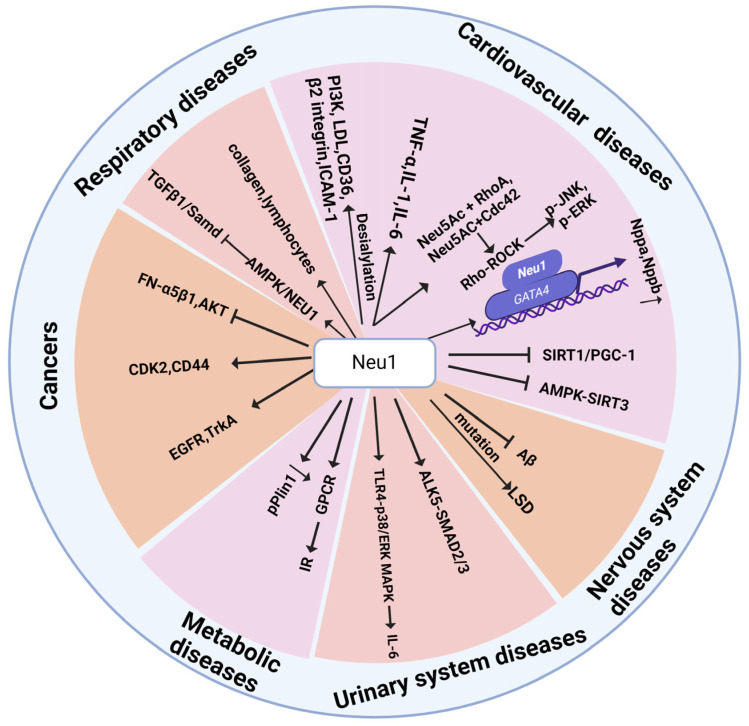
NEU1-regulated signaling pathways implicated in disease pathogenesis across different systems.

**Figure 4:**
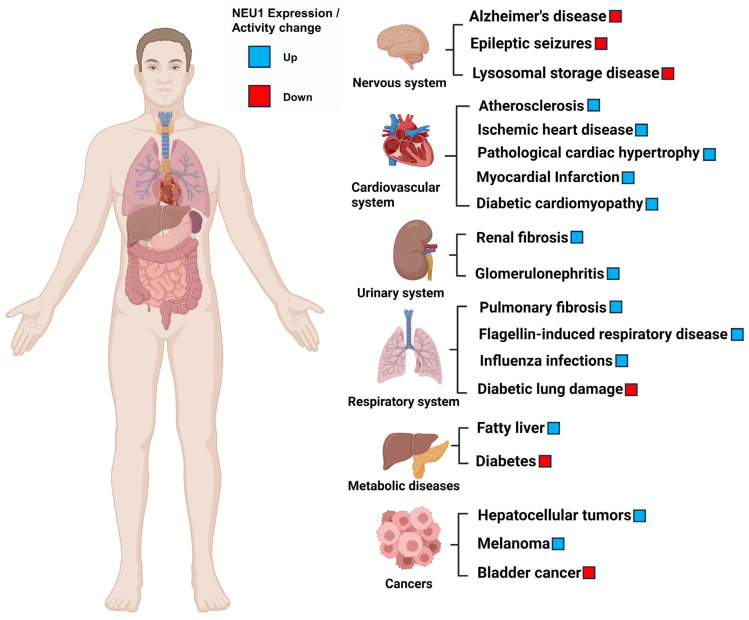
Etiological involvement of NEU1 dysfunction in diseases across various systems.

The diversity of NEU1 substrates generally determines the breadth of NEU1 regulatory effects (Figure 3). NEU1 is fundamentally involved in the coordination of various cellular metabolic behaviors and signaling in vivo, thereby impacting the onset and development of related diseases, such as cardiovascular diseases, neurological disorders, respiratory diseases, hematological diseases, and cancer (Figure 4).

## 3. Pathological Implications of NEU1 across Various Systems

### 3.1. Cardiovascular Diseases

NEU1 plays important physiological roles in maintaining cardiac health. NEU1 is localized to cardiomyocytes, which constitute more than 50% of the adult ventricular region, a proportion much greater than cardiac monocytes and macrophages (~3%) [53]. The dysregulation of NEU1 function has been implicated in cardiovascular pathologies including atherosclerosis [27], vascular plaque inflammation [26], myocardial infarction [54], myocardial hypertrophy [55], heart failure [56], and diabetic cardiomyopathy (DCM) [57].

#### 3.1.1. Atherosclerosis

The endothelial cell surface is amply decorated with sialylation, where Neus, particularly NEU1, works by limiting angiogenesis [49]. Abnormal SA modifications play a crucial role in the development of atherosclerosis through altering the endothelial cell uptake of low-density lipoproteins (LDLs) and leukocyte and platelet adhesion [58]. As an important constituent of LDL, SAs supply the sugar chains in apolipoprotein B (apo B) and in gangliosides. It has been shown that hypo-sialyated LDL particles are more likely to accumulate in the arterial intima and subsequently promote pro-inflammatory events [59]. Demina et al. revealed that NEU1 and NEU3 trigger the initial stages of atherosclerosis characterized by the formation of aortic fatty streaks by desialylating LDL glycoproteins and apoB 100, consequently augmenting their uptake by macrophages through asialoglycoprotein receptor [60]. Elastin-derived peptides (EDPs) constitute another risk factor for the development of atherosclerosis, whose atherogenic effects depend on sialidase activity and the NEU1/PI3Kγ signaling pathway as key regulators of its function in vitro and in vivo [61].

In contexts of macrophage-mediated inflammation, CD36 plays pro-atherogenic roles through its interaction with oxidized low-density lipoprotein (oxLDL), which triggers the signaling cascades of inflammatory responses, oxLDL uptake, and the formation of foam cells, setting in motion the initial stage of atherosclerosis. To elaborate, CD36 has been implicated as at least 50% of the cause of oxLDL uptake by macrophages in both mice and humans [62,63]. Remarkably, CD36 has emerged as a novel interaction partner of plasma membrane NEU1 in macrophages. EDP activities impact the course of atherosclerosis through regulating the levels of the sialylation of CD36, a process largely governed by the ERC and its catalytic subunit, NEU1, to modulate oxLDL uptake in these cells [50]. In addition, ERC reportedly interacts with *β*2 integrin and ICAM-1 through the membrane NEU1 in monocytes and endothelial cells, respectively. The binding of EDP to the ERC results in the modulation of *β*2 integrin and ICAM-1 sialylation levels, which in turn significantly increases monocyte adhesion to endothelial cells and monocyte migration across the endothelium [64].

#### 3.1.2. Ischemic Heart Disease

Recent findings suggest that NEU1 dysregulation is also implicated in ischemic heart disease. The NEU1/*β*-Gal/PPCA complex adversely affects cardiac damage after ischemia/reperfusion (I/R). The selective modulation of this complex has been proposed as a novel therapeutic strategy for reducing the risks of chronic heart remodeling and heart failure. Pathologically, NEU1 influences the trajectory of I/R injury primarily by modulating intercellular communication [24,25,26,27]. For example, three days after the induction of I/R in mice, left ventricles (LV) displayed upregulated Neus activity and elevated NEU1, *β*-Gal, and PPCA expression, whereas mice bearing the phenotype of hypomorphic for neu1 (hNEU1) exhibited lower activities of cofactors PPCA and *β*-Gal [56]. In comparison with wild-type mice, hNEU1 mice were characterized by fewer pro-inflammatory cells, more abundant anti-inflammatory cells, and improved LV function in ischemic LV tissues. Furthermore, mice with cardiomyocyte-specific NEU1 overexpression showed aggravated cardiomyocyte hypertrophy, diminished expression, and the mislocalization of connexin-43 at gap junctions that accompany LV dysfunction. Overall, the upregulation of NEU1 following I/R drives heart failure by promoting inflammation mediated by infiltrating monocytes/macrophages, promoting cardiomyocyte hypertrophy, and impairing gap junction function [56,65].

Typically, ischemic heart disease is accompanied by impaired mitochondrial energy metabolism and oxidative stress. Indeed, serum NEU1 levels are elevated in patients with acute myocardial infarction [66]. NEU1 expression is characteristically upregulated in myocardial infarction (MI) tissues, and is reportedly higher in the mononuclear blood cells of MI patients vis-à-vis healthy controls [27]. Consistently, NEU1 inhibition ameliorates mitochondrial dysfunction, which has attracted much attention in the mechanistic investigation of the onset and progression of MI [67,68,69]. Mitochondria are highly enriched in myocardial tissues due to the high energy requirements of the heart to sustain cardiac functions [70]. Cardiomyocyte-specific NEU1 deficiency can restore cardiac function, thereby improving the conditions of myocardial hypertrophy and interstitial fibrosis. In addition, heart zone-specific NEU1 inhibition prevents the development of cardiac dysfunction and remodeling in patients with chronic MI by ameliorating mitochondrial energy metabolism and dampening mitochondrial oxidative stress in myocardial tissues post-MI. In terms of mechanism, as evidenced in in vivo and in vitro experiments, the genetic knockdown of NEU1 ameliorates cardiomyocyte injury by modulating SIRT1/PGC-1 dependent signaling, eventually enhancing mitochondrial biogenesis and function [54].

The elevations of *N*-acetylneuraminic acid (Neu5Ac) have been observed in plasma during the progression of coronary artery diseases (CADs). Such Neu5Ac elevations precipitate myocardial injury by activating the Rho-ROCK signaling pathway by binding to RhoA and Cdc42, and by enhancing the phosphorylation of JNK and ERK [71]. Silencing NEU1, which is responsible for regulating Neu5Ac generation, ameliorates oxygen/glucose deprivation-induced injury in cardiomyocytes and in ligation/isoprenaline-induced myocardial ischemia injury in rat models.

#### 3.1.3. Pathological Cardiac Hypertrophy

In an experimental model of cardiac hypertrophy and in patients with hypertrophic cardiomyopathy, NEU1 was found to be highly expressed in hypertrophic hearts. Treatment with isoprenaline, a prototypical *β*-adrenoceptor agonist, rapidly increases NEU1 activity in rat cardiomyocytes and immortalized H9c2 myofibroblast cells [72]. Meanwhile, in experiments involving cardiomyocyte-specific NEU1 knockout and NEU1 overexpression, it was found that cardiomyocyte-specific NEU1 knockout significantly alleviates pathological myocardial hypertrophy in the aortic arch constriction (TAC) and isoproterenol (ISO) stimulation models [73], whereas NEU1 overexpression exacerbates TAC-induced pathological myocardial hypertrophy, which suggests that NEU1 critically drives cardiac hypertrophy. Furthermore, NEU1 was demonstrated to translocate into the nucleus under stimulation by pressure overload, and selectively binds to GATA4 upon nuclear translocation [74]. Subsequently, this leads to the transcriptional upregulation of Nppa (ANP) and Nppb (BNP) [75], which are the canonical cardiac hypertrophy-related genes.

Elsewhere, C-09, a compound that forms hydrogen bonds with NEU1, was experimentally shown to improve pathological cardiac hypertrophy by targeting NEU1 [76,77]. C-09 improves mammalian cardiac function by inhibiting NEU1 enzyme activity. These findings generally corroborate the idea that compound C-09 can ameliorate pathological cardiac hypertrophy by pharmacologically targeting NEU1 [78].

#### 3.1.4. Diabetic Cardiomyopathy

In diabetes-related contexts, NEU1 deficiency has been shown to be beneficial toward diabetic cardiomyopathy in mice. Diabetic cardiomyopathy is mechanistically linked to cardiac dysfunction, oxidative stress, fibrosis, and inflammation [79]. NEU1 deficiency attenuates these negative effects within the heart by activating AMPK-SIRT3 signaling, which in turn improves cardiac functioning by boosting energy production and blunting oxidative stress. Additionally, NEU1 inhibition reduces local inflammation and cardiac fibrogenesis, which takes the form of the excessive accumulation of connective tissues in the heart [57].

### 3.2. Nervous System Diseases

The cell-surface carbohydrates of the central nervous system (CNS) tissues are generally sialylated, which modulates the behavior of neurons and glial cells. Sialylation levels are dynamic and fluctuate during brain development. Aberrant changes in sialylation and abnormal alterations in signal transduction involving the excitatory neurotransmitter glutamate have been observed in neurological disorders and during the inflammatory response of microglia [80,81].

#### 3.2.1. Neuroinflammation

When microglia are exposed to lipopolysaccharide (LPS), the translocation of NEU1 to the cell surface occurs, where NEU1 desialylates TLR4, followed by a reduction in Siglec-E binding and the enhancement of the inflammatory activation of microglia. Thus, cell-surface NEU1 has been proposed as a potential drug target for mitigating neuroinflammation [82]. NEU1 has also been reported to be elevated in retinal Müller glial cells (RMG) in inflammatory lesions, implicating NEU1 as a novel activation marker for inflammatory RMG [83].

#### 3.2.2. Alzheimer’s Disease

NEU1 is strongly implicated in Alzheimer’s disease (AD) pathogenesis, where it regulates amyloid precursor protein (APP) metabolism through desialylation. AD is veritably the predominant neurodegenerative disease leading to dementia. AD pathology is cardinally marked by the accumulation of A*β* (Amyloid-*β*, a proteolytic product from APP processing), which in turn fuels the formation of amyloid plaques and neurogenic fibril tangles of tau protein (NFTs) [84,85,86]. Mechanistically, this pathologic feature is linked to impaired autophagy in microglia/macrophage [87], phenotypically manifested as impaired A*β* clearance and the elevated aggregation of amyloid plaques in AD development. Experimentally, NEU1 deficiency in mice induces a spontaneous phenotype of AD-like amyloidosis, while the overexpression of NEU1 is conducive to a reduction in amyloid plaques [22]. Other studies have found that NEU1 signaling can promote macrophage polarization in the M2 state, thereby reducing the formation of toxic oligomers. NEU1 is, therefore, deemed a target with therapeutic potential in AD treatment [55,88].

#### 3.2.3. Epileptic Seizures

The pathogenic homozygous mutation of c.544A>G in exon 3 of the NEU1 gene leads to the development of generalized tonic/clonic seizures with teenage onset, followed by progressive visional decline and limb myoclonus [89]. In animal work modeling pentylenetetrazol-induced seizures, it was demonstrated that sialidase activity (38.14%) is primarily localized to the brainstem, followed by the cerebellum (28.58%) and the cerebral cortex (11.38%), while minimal sialidase activity resides in the hypothalamus (2.88%) [90]. Interictal spikes arising from the synchronous, paroxysmal depolarization of neurons pivotally influence the initiation of epileptic seizures. Intuitively, reduction in interictal spikes should enable the resolution of epileptic attacks. A rational approach to achieving this effect was proposed in a study of sialidase injections in epileptic rat brain [91]. Since the enzyme sialidase regulates the amount of negative charge on cellular membranes, its administration causes a depolarizing shift, eventually lowering the likelihood of unwanted neuronal firing and synchronization.

#### 3.2.4. Neuronal Impairment

In some neural progenitor cell and neuronal disease models, the disease phenotypes were characterized by reduced Neu activity, the accumulation of sialyl-oligoconjugates, and lysosomal amplification. Two aberrant alterations of glutamate efflux release defects and α-amino-3-hydroxy-5-methyl-4-isoxazolepropionic acid receptor (AMPAR)-mediated increases in Ca^2+^ influx have been identified. Both of these abnormal changes are associated with neuronal damage [92,93]. The overexpression of the wild-type NEU1 gene restored these abnormalities, suggesting that Neu deficiency is a likely cause for the functional impairments of neuronal dysfunction. Proteomic analyses revealed significant reductions in SNARE proteins and glycolytic enzymes in synaptosomal fractions, with the concomitant downregulation of ATP production. Bypassing glycolysis by treating pyruvate (the final metabolite of the glycolytic pathway) ameliorated synaptosomal ATP production and exocytosis [94]. AMPAR and *L*-type voltage-dependent Ca^2+^ channel (VDCC) subunits were upregulated in dysfunctional neurons, while the antagonists of AMPAR and *L*-type VDCC restored AMPAR-mediated Ca^2+^ overload [93,95,96].

### 3.3. Lysosomal Storage Disease

Sialidosis and galactosialidosis (GS) are two autosomal recessive lysosomal storage diseases (LSDs) associated with the genetic mutations of NEU1 and PPCA, respectively [97,98]. These diseases most commonly occur in adolescents, whose clinical manifestations include visual disturbances and mild neurological symptoms [99]. As a result of NEU1 mutations in sialidosis, for example, the degradation of sialylated glycoproteins becomes dysregulated, accompanied by an increase in high-SA metabolites. GS arises as a result of autosomal recessive NEU1 deficiency, etiologically linked to a primary defect in the PPCA gene [99,100]. Both LSDs are characterized by the progressive accumulation of sialylated glycoproteins and oligosaccharides in the lysosomes across multiple cell types. However, there remains relatively scarce research on LSDs, with virtually no specific drugs for disease intervention.

At present, the development of targeted therapies for GS is an area of active pursuit, with several promising therapeutic regimens being investigated in *Ctsa*^−/−^ mice for translational applications in patients. These include bone marrow (BM)-mediated in vitro gene therapy, enzyme replacement therapy (ERT) [101], and adeno-associated virus (AAV)-mediated in vivo gene therapy. Notably, AAV has become an in vivo gene transfer system of choice for the treatment of LSDs by virtue of its infectious efficacy, broad indications, and safety profiles. Multiple AAV serotypes have been designed that are pleiotropic for specific cell populations (e.g., hepatocytes and neurons) and have been tested in LSD patients and animal models. It was demonstrated that a self-complementing AAV2/8 vector (scAAV2/8-*CTSA*) can be employed to control the hepatic expression of human *CTSA* transgenes driven by a liver-specific enhancer/promoter combination [102]. High doses (2 × 10^13^ gc/kg) of scAAV2/8-*CTSA* treatment in *Ctsa*^−/−^ mice were tested to assess the biodistribution, long-term efficacy, and safety of AAV, including possible inflammation and tumors, and the presence or absence of neutralizing antibodies. The reported findings suggest that in addition to the correction of histopathological, biochemical, and clinical phenotypes, the systemic administration of high doses of recombinant vectors did not cause such observable adverse effects as hepatotoxicity, immune responses, or hyperplasia. Refined toxicity studies on AAV-mediated gene therapy should contribute to the development of safer and more effective orphan treatments for LSDs.

In a study, two scAAV2/8 vectors expressing human NEU1 and its chaperone protein, PPCA, were injected into *Neu1*^−/−^ mice [103]. It was found that in most tissues, including the brain, heart, muscle, and visceral organs, NEU1 activity was restored to varying degrees with a reversal of sialyl-oligosacchariduria. Therefore, AAV-mediated gene therapy appears a suitable treatment for sialidosis and other diseases associated with low NEU1 expression [20,103,104]. Gene therapy approaches may enable stable, long-lasting therapeutic correction. Unfortunately, these treatments may not be available for clinical use anytime soon as the number of eligible patients remains low. Interestingly, dietary supplementation with betaine, a naturally occurring amino acid derivative, increased levels of mutant NEU1 and resolved the oligosacchariduria in *Neu1*^−/−^ mice. The findings suggest that selectively balanced use of non-traditional dietary compounds in traditional therapeutic approaches may be beneficial in the treatment of sialidosis and should be generally applicable to all attenuated LSD [105]. In addition, combinatorial bibliometric and machine learning methods have proven useful for elucidating possible links between disease targets and accessible bioactivity data, thereby integrating small molecule data and building drug discovery models for rare diseases related to NEU1. Such approaches are anticipated to accelerate the discovery of new compounds that are potential drug candidates [106].

### 3.4. Respiratory Diseases

A linkage of Neus, particularly NEU1, with lung pathologies has been proposed [107]. NEU1 is abundantly expressed in the three major cell types of the human lung, namely: human airway epithelial cells (HAECs), human pulmonary microvascular endothelial cells (HPMECs), and human lung fibroblasts (HLFs) [46,108]. NEU1 activity is needed to sustain a variety of biological processes in the respiratory system, while NEU1 deficiency or dysfunction has been implicated in the abnormal metabolism of alveolar surface-active substances, resulting in respiratory diseases [30].

#### 3.4.1. Pulmonary Fibrosis

In idiopathic pulmonary fibrosis and bleomycin-challenged mice, NEU1 expression is increased significantly with respect to that of the controls, affecting gene expression profiles in lung microvascular endothelial cells. In contrast, NEU2, NEU3, and NEU4 were found to be expressed at far lower levels [109]. The overexpression of NEU1 disrupts endothelial capillary-like formation. It additionally promotes lung collagen deposition, lymphocytosis, and fibrosis, thereby aggravating lung injury. For bleomycin-induced pulmonary fibrosis in mice, the selective NEU1 inhibitor C_9_-butyl-amide-DANA (C_9_-BA-DANA) strongly reduces the lung inflammation index and fibrosis index compared with untreated controls. C_9_-BA-DANA was found to be as efficacious as the broad-spectrum neuraminidase inhibitor, 2,3-dehydro-2-deoxy-*N*-acetylneuraminic acid (DANA) [109,110].

#### 3.4.2. Flagellin-Induced Respiratory Disease

Airway epithelia express sialylated receptors for the recognition of exogenous danger signals. NEU1 occurs as the predominant NEU in human respiratory epithelial cells and lung microvascular endothelial cells. It was found that the Gram-negative pathogen *Pseudomonas aeruginosa* (Pa) expresses a type of adhesion protein, flagellin, which binds to the outer structural domain (ED) of mucin 1 (MUC1). The NEU1-mediated desialylation of MUC1-ED increases its shedding into the airway lumen, promoting the generation of a soluble, highly viscous decoy receptor for Pa [46]. Further studies revealed that C9-BA-DANA, a specific inhibitor of NEU1, dose-dependently inhibited the flagellin-induced desialylation, detachment, and adhesion of MUC1-ED, and attenuated the NEU1-mediated inhibition of cell migration and disruption of capillary-like vascularization [110].

#### 3.4.3. Influenza Infections

The glycoprotein hemagglutinin (HA) and Neus occurring on the surface of influenza viruses co-determine viral typing and infection efficiency, which have been one of the research foci on human infectious diseases due to the unpredictable and highly mutable nature of influenza viruses [111,112]. Most anti-influenza medications currently available at the clinic are glycosidase inhibitors, which include tamiflu and zanamivir [73,74]. Tamiflu, whose main ingredient is oseltamivir phosphate [113], has likewise proved effective in patients with avian influenza. Hence, in the contexts of influenza virus maturation and transmission, the roles of NEU1 as a neuraminidase highly expressed in human tissues deserve particular attention. NEU1 has been proposed as a potential therapeutic target for COVID-19, and against viral pathogens in future coronavirus pandemics, on the basis that NEU1 inhibitors can limit SARS-CoV-2 replication in COVID-19 patients [114,115].

#### 3.4.4. Diabetic Lung Injury

In patients with diabetes, complications profoundly impact outcomes in terms of mortality and disability, though diabetic lung injury has remained quite insufficiently examined. Recent evidence suggests that *Coptis chinensis* inflorescence extracts (CE) exert hypoglycemic effects, while its isolated components berberine (BBR) and linalin (LIN) can improve metabolic abnormalities, and reduce lung inflammation in diabetic mice. In terms of mechanisms, LIN or BBR effectively alleviates diabetic lung injury by modulating the AMPK/NEU1-mediated signaling to restrict hyperglycemia-induced alveolar epithelial/mesenchymal transition and consequently lower TGF-*β*1 levels [116].

### 3.5. Diseases of the Urinary System

NEU1 figures are important in the pathogenesis of urinary system diseases, notably kidney diseases [117,118].

As NEU1 regulates the maintenance and repair of glomerular filtration membranes, NEU1 deficiency can lead to the damage of these membranes, subsequently paving the way for the development of urological disorders, such as glomerulonephritis, nephrotic syndrome, etc. [119,120]. NEU1 defects result in the aberrant expression or processing of megalin associated with reabsorption, leading to increased albumin levels in the urine [118].

#### 3.5.1. Renal Fibrosis

As evidenced by studies with clinical samples and animal and cellular models of renal fibrosis, NEU1 is significantly upregulated in renal fibrotic conditions. Highly expressed NEU1 localized predominantly in renal tubular epithelial cells. Within models of unilateral ureteral ligation (UUO) and folic acid (FA) stimulation, NEU1 knockout was effective in inhibiting epithelial/mesenchymal transition [121], inflammatory cytokine production, and collagen deposition, thus alleviating renal fibrosis and renal injury. On the other hand, the overexpression of the NEU1 gene exacerbated progressive renal fibrosis. Evidence suggests that NEU1 binds to the amino acid residue 160–200 region of ALK5 to stabilize ALK5, which consequently leads to SMAD2/3 activation [122]. In drug screens, the plant-derived compound salvianolic acid B from *Salvia miltiorrhiza* binds strongly to NEU1, setting off signaling cascades that protect mice from renal fibrosis in a NEU1-dependent manner [123,124].

#### 3.5.2. Glomerulonephritis

As a regulator of cytokine release in autoimmunity, NEU1 is etiologically significant to the pathogenesis of lupus glomerulonephritis [125]. Lupus glomerulonephritis occurs as a severe form of complication in systemic lupus erythematosus (SLE) [126], whose development encompasses an array of dynamic factors including the activation of inflammatory responses and glomerular injury. NEU1 is known to be implicated in some of the key steps in these processes. For instance, NEU1 regulates the formation and deposition of immune complexes in cellular events, leading to lupus glomerulonephritis [127]. NEU1 is also responsible for coordinating inflammatory responses in lupus glomerulonephritis, such as the release of tumor necrosis factor-*α* (TNF-*α*) and interleukin-6 (IL-6) [128]. NEU1 activity promotes IL-6 secretion from lupus-prone MRL/lpr primary mouse mesangial cells (MCs) in response to an IgG mimic, and to circulating lupus factors through the TLR4-MAPK p38 or ERK signaling pathway. The latter pathway was deemed a direct mediator of Neu activity, as both IL-6 protein and IL-6 mRNA levels are significantly attenuated via the blockage of Neu activity [129].

In vitro, although renal NEU1 is primarily responsible for mediating cytokine release from MCs, it may not be involved in regulating renal glycosphingolipid (GSL) levels in vivo or influencing the pathogenesis of nephritis in lupus-susceptible mice. Further scrutiny is warranted to clarify the roles and mechanisms of GSL metabolism, sialylated glycans, and NEU1 activity in lupus glomerulonephritis [130].

### 3.6. Cancers

The status of sialylation as glycoprotein modifications on the surface of cancer cells can be exploited as a proxy to distinguish different tumors. As increased ST activity/expression, SA hyper-synthesis, and the differential expression of endogenous Neus are observed in tumorigenesis, it has been proposed that SAs can serve as a potential therapeutic target [23]. Altered NEU1 expression has been linked to the degree of tumor progression in various cancers (Table 2).

#### 3.6.1. Hepatocellular Cancers

NEU1 has emerged as a new biomarker for hepatocellular carcinoma (HCC), one of the deadliest malignancies, which lacks diagnostic efficiency [133]. Recently, it has been found via ONCOMINE analysis that NEU1 has high expression in HCC, with upregulated mRNA and protein levels relative to normal controls. This was thought to result in the increased migration and proliferation of HCC cells. High expression levels of NEU1 were positively correlated with adverse prognosis in HCC patients, which may be attributed to NEU1’s regulation of oncogenic pathways and the inhibition of immune functions [131,132].

#### 3.6.2. Melanoma

Similarly, NEU1 was found to be highly expressed in melanoma samples compared to normal samples. NEU1 undergoes mutations in 18% of melanoma patients. The expression of NEU1 is positively correlated with overall survival in melanoma patients [134]. The expression of NEU1 was found positively correlated with the expression levels of the proliferation markers CDK2 and epithelial/mesenchymal transformation marker CD44, and negatively correlated with the expression of apoptosis markers CASP3 and CASP8. NEU1 expression levels predicate the infiltration of immune cells in melanoma in vivo. Consistently, knockdown NEU1 restricts the in vitro proliferation and migration capacity in melanoma cells, with similar effects in vivo against melanoma progression [134].

#### 3.6.3. Bladder Cancer

In another urological disease context, evidence shows that NEU1 can inhibit the progression of human bladder cancer, mainly by dampening signal transduction mediated by fibronectin/integrin *α*5*β*1 and the Akt signaling pathway, culminating in reduced Akt activation [135,136]. Fibronectin (FN), a high molecular-weight glycoprotein occurring in the extracellular matrix, binds to the transmembrane integrin *α*5*β*1 to regulate cell expansion and migration. Experimentally, it was confirmed that NEU1 overexpression inhibits tumor formation in vivo and in vitro by inhibiting the proliferative and metastasizing tendencies of tumor cells [143].

#### 3.6.4. Pancreatic Cancers

Multiple receptors involved in signaling pathways implicated in cancer progression, such as EGFR, TrkA, and TOLL-like receptors, are associated with NEU1. In pancreatic cancer cells, NEU1 removes sialic acids from EGFR, leading to EGFR dimerization and the activation of pro-survival pathways. The inhibition of NEU1 by oseltamivir phosphate prevents dimerization through steric hindrance. This work demonstrates that increased NEU-1 expression is essential in EGFR signaling, which promotes cancer progression and metastasis [137,138].

Oseltamivir inhibits NEU-1 activity and suppresses intrinsic signaling for the survival of human pancreatic cancer cells with chemoresistance [113,144]. In addition, aspirin and celecoxib inhibit NEU1, modulate EFG-induced growth receptor activation, and induce apoptosis and necrosis in dose- and time-dependent manners [145].

However, in another study, miR-125b expression was shown to be increased in a cell line model of gemcitabine-resistant pancreatic ductal adenocarcinoma (PDAC) in the contexts of EMT and chemoresistance, which lends support to the potential antitumor activity of NEU1 as evidenced by the attenuated expression of NEU-1 in the model [146].

#### 3.6.5. Aggressive Pleomorphic Sarcomas

NEU1 negatively regulates the extracellular actions of lysosomes by cleaving LAMP1, thereby affecting tumor invasiveness. Low expression of NEU1, on the other hand, facilitates the formation of aggressive pleomorphic sarcomas, promoting the expression of epithelial and mesenchymal cell recognition molecules [51]. The NEU1-regulated expression of the lysosomal extracellular factors LAMP1 and myosin-11 characterizes human metastatic pleomorphic sarcoma, suggesting that the lysosomal regulatory pathway is crucial to tumorigenesis and drug resistance [141,142].

#### 3.6.6. Colon Cancer

The assessment of human NEU1 expression in colon cancer by PCR showed that its expression in cancerous tissues was lower than that in adjacent noncancerous mucosa, while the levels of NEU1 activity in the same cancerous tissue appeared to be negatively correlated with the degree of invasion or differentiation [147]. Furthermore, NEU1 overexpression in colon cancer HT-29 cells decreased cell migration and cancer cell invasion. Conversely, NEU1 knockdown increased cell migration and invasion. Additionally, in vivo, liver metastasis was significantly reduced when NEU1 was overexpressed. The authors found that NEU1 was able to reduce *β*4-integrin sialylation, which decreased the phosphorylation of the FAK and ERK1/2 pathways, thereby inhibiting *β*4-integrin-dependent cell migration, invasion, and adhesion [48]. These results suggest that the overexpression of NEU1 is negatively correlated with colon cancer cell invasion.

#### 3.6.7. Other Cancers

For example, the downregulation of the expression of NEU1 in ovarian cancer cells OVCAR3 and SKOV3 promotes apoptosis, inhibits cell proliferation and invasion, and effectively restricts malignant phenotypes of tumors [140]. Likewise, oseltamivir phosphate weakens angiogenesis, growth, and metastasis of triple-negative breast cancer cells in murine models [139]. There are also studies showing that the high expression of NEU1 plays a positive role in cancer inhibition, as illustrated by the inhibited progression of colon and cervical cancers [48,148,149].

Overall, a consistent role of NEU1 in different tumors has not been established, with either too high or too low expression of NEU1 being a driver of divergent effects on different cancers. The expression of this protein shows distinct patterns in different organogenic tumors. Thus, the complexity of its functions in various organs still needs further scrutiny. Restoring the normal levels of NEU1 may constitute a logical strategy for the treatment of tumors.

### 3.7. Metabolic Diseases

The dysregulation of NEU1 is associated with the onset and evolution of metabolic diseases, as NEU1 deficiency can lead to cellular aberrations in glucose, lipid [150], and protein metabolism, which in turn influences the trajectory of metabolic diseases.

#### 3.7.1. Fatty Liver Disease

The expression levels of miR-205 are reportedly decreased in the liver tissues of patients with NAFLD (non-alcoholic fatty liver disease) [151]. In in vivo and in vitro experiments, miR-205 was found to inhibit lipid accumulation in adipocytes and regulate the transcriptional expression of the NEU1 gene. Likewise, miR-205 reduces lipid accumulation in hepatocytes by suppressing the expression of NEU1, thus resolving some of the symptoms of NAFLD [152]. Recent evidence further suggests that NEU1 can inhibit lipolysis via perilipin 1, resulting in lipid accumulation [153,154].

#### 3.7.2. Diabetes

As part of the regulatory machinery for energy metabolism and glucose uptake, it is not surprising that NEU1 is pathologically involved in the development of diabetes. NEU1 engages in the regulation of insulin signaling in multiple ways, including facilitating the initiation of insulin signaling, reversing insulin resistance, and cross-talking with MMP9. SA and its associated metabolic enzymes have emerged as important components of the pathophysiology of type 2 diabetes mellitus (T2DM) [155]. Emerging evidence supports the proposition that NEU1 can serve as a potential drug target for type 2 diabetes, wherein its activation may help rectify insulin resistance and check aberrations in glucose metabolism. While insulin resistance is a cardinal hallmark of T2DM, it ultimately drives a compensatory increase in insulin secretion and *β*-cell hypertrophy [28,29]. Some researchers have suggested that following exposure to a high-fat diet (HFD), mice with 10% NEU1 activity in their tissues develop glucose intolerance and insulin resistance faster than wild-type mice, and suffer other repercussions such as impaired insulin signaling in their tissues. Molecular analyses revealed that NEU1 sets off insulin signaling by inducing the formation of its active dimer via desialylated insulin receptor (IR) [156]. The high expression of NEU1 predictably reverses insulin resistance [47]. Ambroxol pharmacologically reverses the abnormalities of insulin resistance and glucose metabolism in mice through the acute pharmacological induction of NEU1 activation, corroborating that NEU1 can serve as a drug target in T2DM [156].

In addition, T2DM is known to be associated with the elevated expression of MMPs (especially MMP-2 and 9) and increased degradation of elastin and elastin fibers that leads to the production of elastin-derived peptides (EDPs) [157,158,159,160]. The binding of insulin to its extracellular *α*-receptor subunit pivotally induces a conformational change in the receptor, which sets in motion G-protein coupled receptor (GPCR) signaling, whereas NEU1 hydrolyzes sialic acid residues within the glycan chain of the IR kinase, triggering its activation. Importantly, during MMP9 activation via GPCR signaling, EBP functions as part of a multi-enzyme complex containing the classical *β*-gal/NEU1 and PPCA. Cross-talk between NEU1 and MMP9 seems necessary for insulin-induced IR activation and cell signaling, where IR activation is positively regulated in a manner dependent on GPCR signaling and cell membrane NEU1 sialidase activity. Consistently, GPCR agonists can activate IR in the absence of insulin ligands, a process controlled by NEU1 and neuromodulin B receptors [161].

## 4. Conclusions and Perspectives

In this review, the many nuances of NEU1’s etiological involvement in SA-related human diseases are discussed, which provides exciting hints on the prospect of exploiting NEU1 as an intervention target by selective inhibitors or activators. In particular, research on NEU1 in recent years has shifted in focus toward NEU1’s multifaceted roles in diverse cancer types, in such contexts as its regulation of tumor development, migration, proliferation, and related signal transduction pathways. However, the specific regulatory mechanisms of NEU1 in tumors remain only partially understood. Abnormal glycosylation modifications, for example, have been observed in cancer tissues, though a unified discourse of interpretations is lacking. NEU1’s mechanistic roles in different cancers remain elusive. Therefore, caution and further scrutiny are needed before arriving at translationally viable NEU1-based pharmacological treatment against cancer.

Elsewhere, the diversity of NEU1 substrates allows NEU1 to participate in multiple signaling pathways, inspiring the innovations of rational drug design and development beyond cancer contexts. Partly due to a lack of structural information about NEU1, until recently no selective inhibitors for NEU1 had been identified. Therefore, when studying the biological roles of NEU1, the broad-spectrum sialidase inhibitor DANA or inhibitors of bacterial or viral NEUs such as zanamivir or oseltamivir have been generally used. Although some studies have utilized zanamivir or oseltamivir to inhibit NEU1 to show therapeutic efficacy, current evidence suggests that bacterial or viral NEU inhibitors do not specifically inhibit human NEU1, and weakly inhibit human NEUs. Thus, it remains controversial as to whether these compounds can be called reliable inhibitors with adequate NEU1 specificity [162]. However, several selective inhibitors of human NEU1 based on DANA scaffold modification have been reported in the literature, including the c9 amino analog of DANA (C9-BA-DANA) [163], c5-hexamethyl-c9-acetylamino, and C5-hexanamido-C9-acetamido-DANA that has a *K*_i_ of 53 ± 5 nM and 340-fold selectivity over other human NEUs [164]. These compounds were tested in vitro and in vivo and found to effectively inhibit the activity of endogenous and ectopically expressed NEU1 [60,109,156,164,165]. In addition, an interfering peptide that selectively blocks plasma membrane NEU1 (mNEU1) salivary acid lyase activity by interfering with its dimerization was reported but not tested in in vivo models [166]. The recent identification of a three-dimensional structure of human NEU1 [42] should help to accelerate the discovery of more targeted inhibitors or agonists to facilitate the management of NEU1-related diseases with improved therapeutic precision.

A good portion of NEU1 studies have focused on its regulatory roles over downstream targets. It seems imperative to expand the scope of the investigation to how NEU1 itself is precisely regulated in dedicated disease contexts. For instance, NEU1 activity is dependent on the PPCA protein; clarifying the regulation of NEU1 activity by PPCA could potentially inform therapeutic approaches. Likewise, given the functional relatedness between NEU1 and MMP, exploration of the identification or design of dual-targeted NEU1-MMP inhibitors could be another worthwhile avenue of therapeutics development. Collectively, NEU1 research represents an exciting and fast-evolving enterprise that should continue to supply insights into the intimate links among SA metabolism, cell surface receptor modifications, and disease pathogenesis.

## Figures and Tables

**Table 1:** Characteristics of mammalian neuraminidases (Neus).

	NEU1	NEU2	NEU3	NEU4
**Localization**	Lysosome and plasma membrane	cytosol	plasma membrane	endoplasmic reticulum, lysosome, and mitochondria
**Substrate**	Oligosaccharides and glycopeptides	oligosaccharides, glycoproteins, and gangliosides	gangliosides	oligosaccharides, glycoproteins, and gangliosides
**Tissue distribution**	kidney, pancreas, skeletal muscle, liver, lungs, cardiomyocytes, placenta, and brain	muscle tissues	adrenal gland, skeletal muscle, heart, testis, and thymus	brain, skeletal muscle, kidneys, heart, placenta, and liver
**Physiological** **function**	exocytosis, immunity, phagocytosis, lysosomal degradation, and elastic fiber assembly	the differentiation of myofibroblasts and nerve cells	neuronal cell differentiation, apoptosis, and adhesion	neuronal cell differentiation, apoptosis, and adhesion
**Associated disease**	sialidosis, neurodegenerative disorders, cancer, diabetes, and cardiovascular diseases	cancer	neurodegenerative disorders	neurodegenerative disorders and cancer
**References**	[31,32]	[8,33]	[34,35]	[36,37,38]

**Table 2:** Examples of NEU1 implications in cancers.

Diseases	NEU1	Biological Effects	References
**Hepatocellular cancer**	Up	Correlation between higher mRNA and protein expression in cancer cells and shortened survival, and the promotion of hepatocellular carcinoma cell proliferation and migration.	[131,132,133]
**Melanoma**	Up	NEU1 expression was positively correlated with tumor cell proliferation markers and epithelial/mesenchymal transition markers, and negatively correlated with apoptosis markers.	[134]
**Bladder cancer**	Down	NEU1 overexpression enhanced apoptosis and reduced the proliferation of bladder cancer cells.	[135,136]
**Pancreatic cancer**	Up	Interaction with EGFR promoting cancer progression and metastasis.	[137,138]
**Breast cancer**	Up	Plays a role in proliferation, apoptosis and epithelial/mesenchymal transition.	[139]
**Ovarian cancer**	Up	Plays a role in cell proliferation, migration, invasion, and cancer metastasis.	[140]
**Colon cancer**	Down	NEU1 suppressed cell migration, invasion, and adhesion in vitro.	[48]
**Aggressive pleomorphic sarcomas**	Down	Low expression of NEU1 facilitates the formation of aggressive pleo-morphic sarcomas, promoting the expression of epithelial and mesenchymal cell recognition molecules.	[51,141,142]
